# Experimental Evaluation of the Mechanical Properties and Microstructure of Cemented Paste Backfill Containing Rheological Modifier

**DOI:** 10.3390/ma19204285

**Published:** 2026-10-11

**Authors:** Bo Gao, Jiajian Li, Huan Zhang, Yunpeng Kou, Guangbo Li, Qiang Li, Weidong Song

**Affiliations:** 1 School of Resources and Safety Engineering, University of Science and Technology Beijing, Beijing 100083, China; 2 Shandong Gold Group Co., Ltd., Jinan 250100, China; 3 China ENFI Engineering Corporation, Beijing 100038, China; 4 Backfill Engineering Laboratory, Shandong Gold Mining Technology Co., Ltd., Yantai 261400, China

**Keywords:** cemented paste backfill, rheological modifier, nuclear magnetic resonance, computed X-ray tomography, microstructure

## Abstract

The macroscopic mechanical properties of cemented paste backfill (CPB) are closely related to its microstructural evolution. However, the mechanism by which rheology modifier influences the mechanical properties of CPB while improving the flowability of the backfill slurry remains unclear. This study investigated the effects of different cementitious material-to-tailings ratios (c/t) (1:6, 1:8, 1:10) in CPB containing a rheological modifier on the mechanical properties and microstructure at various curing ages (7 d, 14 d, 28 d). Strength parameters and failure characteristics of CPB were determined through uniaxial compression tests and direct shear tests. Pore structure, crack analysis, and microstructural characterization were conducted using nuclear magnetic resonance (NMR), computed tomography (CT), and scanning electron microscopy (SEM/EDS). The results of the study indicate that both uniaxial compressive strength and shear strength increase with higher c/t ratios and longer curing periods. The addition of rheological modifier resulted in a 0.9% to 31.5% reduction in uniaxial compressive strength. This negative effect was more pronounced at low c/t ratios and during early-stage curing. However, the effect on shear strength is twofold. Cohesion decreased significantly, while the angle of internal friction increased slightly. Therefore, the strength-reducing effect of the rheological modifier is somewhat mitigated under high normal stress conditions. Analysis of the stress–strain curves indicates that the rheological modifier increased the deformation during the pore consolidation stage and reduced the slope during the linearly elastic stage. However, it slowed the post-peak failure rate, suggesting that it improved the ductility characteristics of CPB to a certain extent. CT analysis showed that the rheological modifier increased the extent of crack damage and the fractal dimension, but did not alter the overall tensile-shear composite failure mode. NMR and SEM analyses further confirmed that the rheological modifier increased the number of micropores, but did not alter the type or elemental composition of the hydration products. This study elucidates the mechanisms by which rheological modifier influences the macroscopic mechanical behavior, pore structure, crack evolution, and microstructure of CPB. It provides a theoretical basis for the optimization of CPB mix designs and the engineering application of rheological modifier.

## 1. Introduction

As shallow mineral resources gradually deplete, the development of deep mineral resources has become an inevitable trend in the field of mining engineering [1,2,3]. The high ground stress, high water pressure, and complex mining-induced stress environments encountered in deep mining impose even more stringent requirements on the mechanical stability of backfill [4,5,6]. Backfill mining has become the preferred method for the safe extraction of deep mineral resources due to its ability to effectively control rock pressure, reduce surface subsidence, improve ore recovery rates, and utilize industrial solid waste [7,8]. Cemented paste backfill (CPB) technology, with its outstanding advantages such as high strength, low bleeding rate, and excellent pipeline transportability, has been increasingly widely adopted in metal mines both domestically and internationally [9,10].

The mechanical properties of CPB are key indicators that determine the quality of mining area backfill and its long-term service stability. These properties are jointly influenced by multiple factors, including the type and amount of cementing material, the physicochemical properties of tailings, and the curing age [11,12,13]. In recent years, numerous scholars have conducted extensive research on the influence of tailings particle size distribution, the quantitative relationship between the cementitious materials at tailings ratios (c/t) and curing age on strength development, and the failure mechanisms of backfill under uniaxial compression and shear loads. This research has provided an important theoretical foundation for the optimization of mix designs and strength prediction for CPB [14,15,16]. For example, Tang et al. investigated the mechanism by which sodium sulfate activators regulate the formation of hydration products and the strength development of the paste [17]. Liu et al. partially substituted cement with fly ash from municipal solid waste incineration in the CPB and found that its active SiO_2_ and Al_2_O_3_ promote the formation of cementitious hydration products, resulting in a denser matrix structure [18]. Fang et al. conducted experimental studies on the reinforcing effects of steel fibers and basalt fibers on the tensile properties of CPB and determined the optimal reinforcement efficiency at a 0.6% blend ratio [19]. Bai et al. prepared a novel CPB using bauxite tailings, red mud, and fly ash, systematically revealing the influence of red mud content on mechanical properties [20].

However, as the transport distance of backfill pipelines increases and the backfill ratio rises, ensuring that the slurry maintains good flowability, resistance to segregation, and pumpability under high-concentration conditions has become one of the bottlenecks limiting the further adoption of CPB technology [21,22,23]. To improve the rheological properties of backfill slurries, rheological modifier has gradually been introduced into the field of CPB [24,25,26]. However, existing studies have largely focused on the effects of rheology modifiers on the pumping performance indicators of fresh slurry, such as yield stress, plastic viscosity, and slump [27,28,29]. Research on the effects of rheology modifiers on the mechanical properties and microstructural evolution of hardened grout is still very limited [30,31,32]. In particular, there is a lack of systematic experimental verification and mechanistic explanations regarding whether rheology modifiers, while improving the flowability of the grout, have negative effects on the formation of hydration products, the development of pore structure, and crack propagation behavior, as well as how these microscale changes translate into differences in macroscopic mechanical properties.

Based on the above analysis, this study utilized tailings from a gold mine in Shandong Province and a Type C cementitious material—specifically, the essential paste component—developed in-house by the mine for backfilling. The study investigated the evolution of mechanical properties and microstructural characteristics of the CPB—prepared using different c/t ratios of 1:6, 1:8, and 1:10—at various curing ages (7 d, 14 d, and 28 d) when treated with a Type K739 rheology modifier. By obtaining the strength parameters and failure modes of the backfill through uniaxial compression and direct shear tests, and combining these with NMR pore structure analysis, 3D CT crack reconstruction, and SEM/EDS microstructural and elemental analysis, this study aims to elucidate the mechanism by which rheology modifiers influence the mechanical properties of CPB. It seeks to provide a theoretical basis and experimental support for the optimization of mix designs for high-concentration CPB and the engineering application of rheology modifiers.

## 2. Materials and Methods

### 2.1. Materials

**Figure 1:**
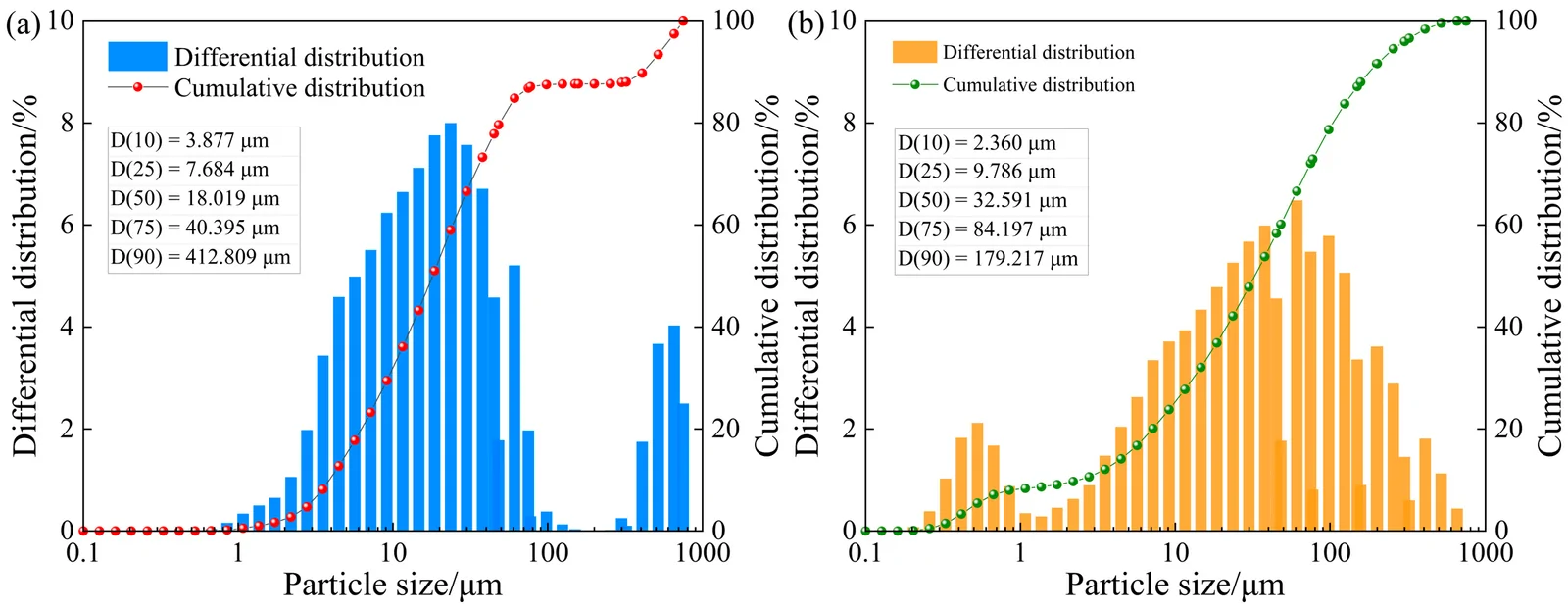
Particle size distribution of raw materials: (**a**) tailings; (**b**) binding material.

The raw materials used in this study were sourced from a gold mine in Shandong Province. The tailings were obtained from the on-site ore processing plant and appear as a grayish-white, fine powder. The cementitious material used is a Type C cementitious material independently developed by the mine. It is primarily composed of industrial by-products such as granulated blast furnace slag, blended with a small amount of silicate cement clinker and an alkaline activator. This specialized cementitious material is designed to enhance the early-stage strength development of the grout backfill, which is critical to the stability of the backfill in underground mining areas. Municipal tap water from the mine was used as the mixing water. Particle size distribution tests and X-ray fluorescence spectroscopy analyses were conducted on the tailings and cementitious materials. The particle size distribution was determined using a laser diffraction particle size analyzer (Mastersizer 3000, Malvern Panalytical, Malvern, UK). Deionized water was used as the dispersion medium. This was supplemented with a 3 min ultrasonic pretreatment to ensure complete dispersion of agglomerated particles. The test results are shown in Figure 1. As can be seen from the figure, the particle size distribution of the tailings spans a wide range and contains a large number of ultrafine particles (D(10) < 4 μm). This broad particle size distribution is conducive to achieving a high bulk density of solids. Fine-grained tailings possess a high specific surface area. To adjust the workability of the grout, 0.3% of a rheology modifier was added during the experiments. The rheological modifier used is the K739 type, manufactured by Shandong Nuoer Biotechnology Co., Ltd., Dongying, Shandong, China. The rheological modifier is a white powder-like solid. The molecular structure of the product was characterized by Fourier-transform infrared spectroscopy (FTIR). The spectrum shows characteristic absorptions at 2876 cm^−1^ (methylene C–H stretching), 1722 cm^−1^ (ester carbonyl C=O stretching) and 1059–1144 cm^−1^ (C–O–C stretching of PEO side chains), confirming the presence of ester linkages and grafted PEO side chains in the copolymer. The overall fineness of the cementitious material is much finer than that of the tailings; it can effectively fill the micro-pores between tailings particles while providing a larger surface area for hydration reactions, thereby promoting early strength development.

**Figure 2:**
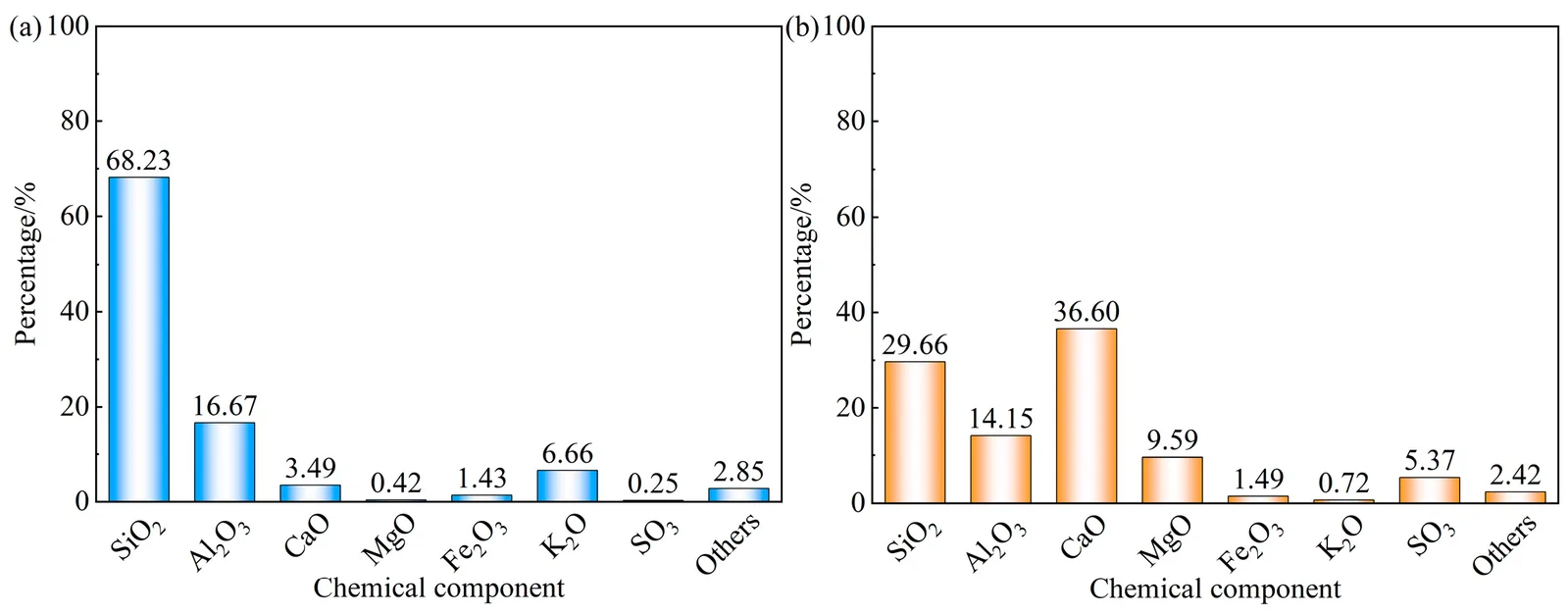
Chemical components of raw materials: (**a**) tailings; (**b**) binding material.

The content of major elemental oxides in tailings and cementitious materials was quantitatively analyzed using an X-ray fluorescence spectrometer (Axios, Panalytical, Eindhoven, The Netherlands). To eliminate the effects of mineral heterogeneity and particle size on the chemical composition analysis, tailings and cementitious material samples were subsampled using the conical quartering method to obtain representative samples. The sub-samples were then finely ground in an agate mortar until they passed a 200-mesh sieve (i.e., <75 μm) to ensure complete homogenization. The instrument parameters for XRF analysis were as follows: scanning speed of 300°/min, X-ray tube voltage of 60 kV, and tube current of 140 mA. The chemical composition of the tailings consists primarily of silicon dioxide and aluminum oxide, along with a certain amount of potassium oxide; the calcium oxide content, however, is relatively low. The main components of Type C cementitious material are calcium oxide, silicon dioxide, aluminum oxide, and magnesium oxide. The high CaO content in the cementitious material helps create a strongly alkaline environment in the pore solution, which is crucial for the hydration of the cementitious material itself. The complete oxide compositions of the two materials are shown in Figure 2.

### 2.2. Specimen Preparation

In this study, three c/t ratios were designed: 1:6, 1:8, and 1:10. The solid content of cementitious material, tailings, and rheological modifiers was fixed at 76% for all test specimens. Add the rheological modifier at a rate of 0.3% of the total mass of the tailings. Among them, the 0.3% addition amount was determined based on the preliminary experiments. In this study, all percentages are expressed as mass percentages; volume percentages were not used. Additionally, to clarify the mechanism of action of the rheology modifier, blank control specimens without the rheology modifier were prepared for each c/t ratio. The sample preparation protocol is shown in Table 1.

**Figure 3:**
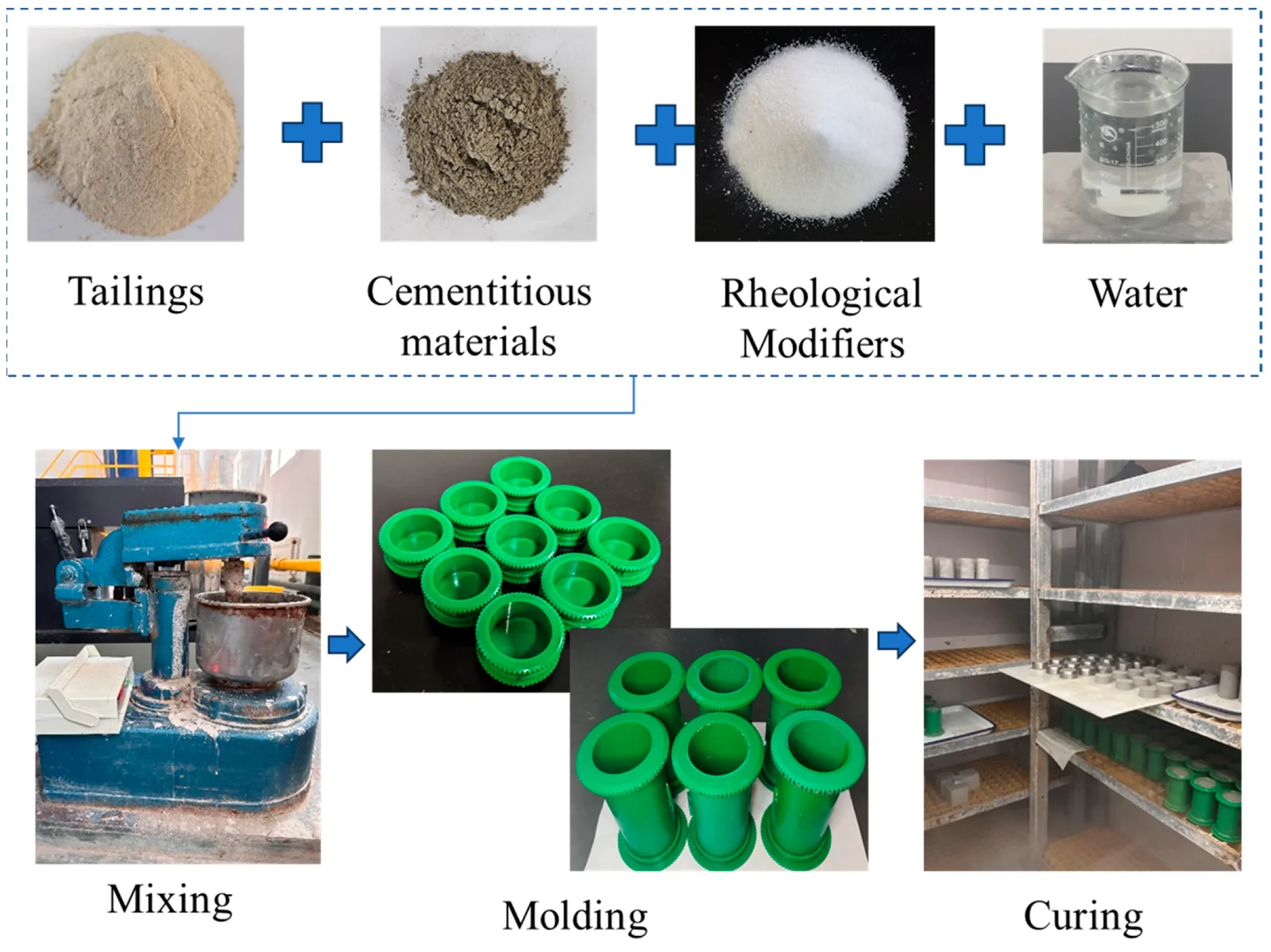
Experimental sample preparation flowchart.

The test specimen preparation process is as follows: First, accurately weigh the tailings, cementitious materials, water, and rheology modifier according to the design mix ratio. Pour the weighed tailings and cementitious materials into a JJ-5 cement mortar mixer (Wuxi Jianyi Instrument Machinery Co., Ltd., Wuxi, China) and dry-mix for 3 min to ensure the two solid materials are thoroughly and uniformly blended. Fully dissolve the rheology modifier in the mixed water until a uniform solution is obtained. Then, pour the resulting solution into the pre-mixed dry solid. Continue to stir for 3 min to ensure that the rheology modifier is evenly distributed in the fresh slurry. After mixing, the fresh slurry is poured into a cylindrical mold with an inner diameter of 50 mm and a height of 100 mm for uniaxial compression testing. Simultaneously, it is poured into a cylindrical mold with an inner diameter of 50 mm and a height of 25 mm for shear testing. After pouring, the molds are vibrated to remove entrapped air bubbles from the slurry and ensure the specimens are densely compacted. The inner walls of the molds were uniformly coated with a thin layer of lubricating oil prior to pouring to facilitate demolding after 48 h. All filled molds were immediately transferred to a standard curing chamber with controlled temperature and humidity for curing. The curing environment was maintained at (20 ± 1) °C, with a relative humidity of no less than 90%. All specimens were cured for 7 d, 14 d, and 28 d, respectively. The specimen preparation process is shown in Figure 3.

### 2.3. Mechanics Performance Testing

**Figure 4:**
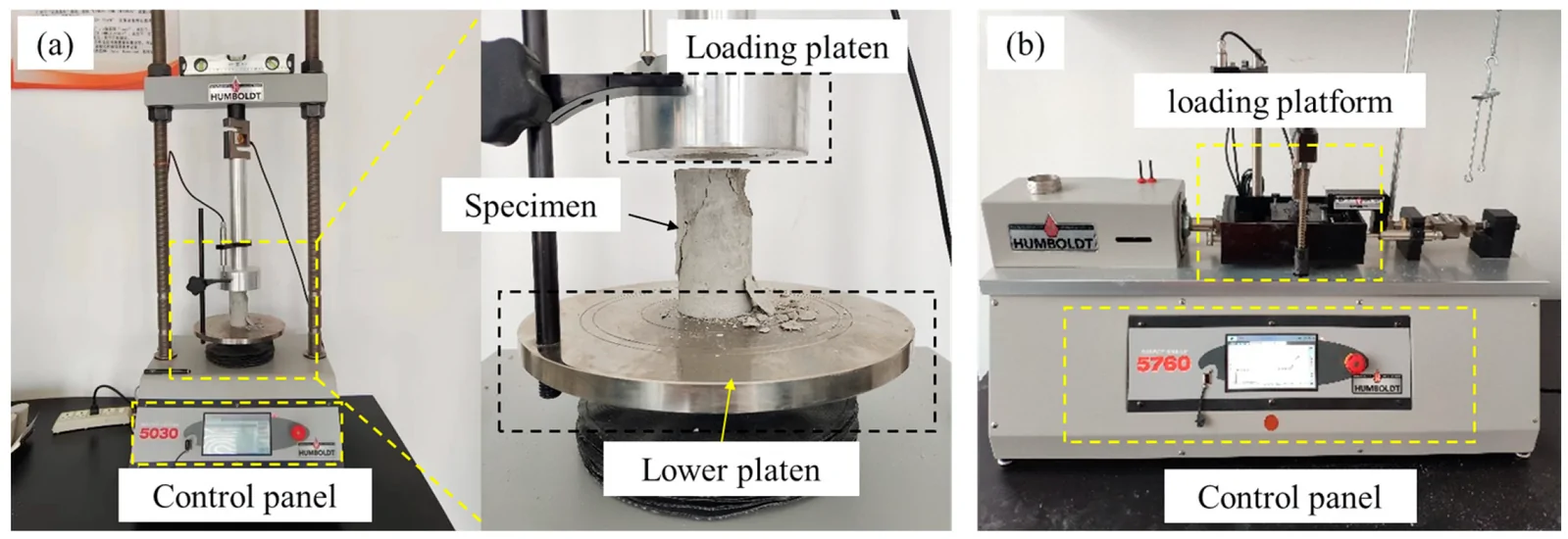
Testing systems used in this study: (**a**) HM-5030.3F uniaxial compression tester; (**b**) HM-5760.3F direct shear tester.

The uniaxial compression test setup is shown in Figure 4a. The uniaxial compression tests were conducted using an HM-5030.3F Marshall uniaxial compression tester manufactured by Humboldt Corporation (Elgin, IL, USA). This instrument primarily consists of a load sensor, a flow sensor, an LCD display, and a loading mechanism. The testing machine has a maximum load capacity of 50 kN, a strain gauge range of 25 mm, and a data acquisition frequency of 10 Hz. Prior to testing, the ends of the cylindrical specimens—cured to the specified ages—were ground flat to eliminate stress concentration effects caused by end irregularities. The test employed a displacement-controlled loading method. The loading rate was set to 1 mm/min. Loading was continued until specimen failure. The instrument automatically recorded the load–displacement curve and the peak failure load. Three parallel specimens were prepared for each mix design and curing age condition. The uniaxial compressive strength was calculated as the average of the three test results, and the standard deviation was calculated to ensure data reliability.

The direct shear test setup is shown in Figure 4b. The direct shear test was conducted using an HM-5760.3F direct shear tester manufactured by Humboldt Corporation (Mfg. Co., Elgin, IL, USA). The instrument primarily consists of a load cell, a horizontal strain gauge, a vertical strain gauge, and a shear box. The main technical specifications are as follows: The maximum horizontal displacement is 50 mm. The maximum horizontal shear force range is 10 kN. The operating voltage is 110/220 V, 50/60 Hz. The rated current is 6.5 A. The test used cylindrical specimens measuring φ50 mm × 25 mm. These specimens were cast from the same batch as the uniaxial compression specimens. The inner diameter of the shear box matches the diameter of the specimen to ensure there is no lateral clearance after the specimen is placed. During the test, a constant normal load is first applied in the vertical direction, followed by uniform horizontal movement of the shear box at a shear rate of 0.5 mm/min until the specimen fails along the predetermined shear plane. During the shear process, the instrument automatically collects data on horizontal shear force and horizontal displacement and records the peak shear force. Three parallel specimens are prepared for each mix design and age condition, and the direct shear strength is calculated as the average of the three test results.

In the direct shear test, five different normal stress levels were applied: 100, 150, 200, 250, and 300 kPa. The shear strength data were analyzed using the Mohr–Coulomb failure criterion. For each set of data, the average shear strength at the five normal stress levels was plotted against the normal stress, and a linear regression analysis was performed using Origin 2025.

### 2.4. NMR Testing

To investigate the evolution of the pore structure in early-stage CPB as a function of rheology additive incorporation and c/t, nuclear magnetic resonance (NMR) technology was used to analyze the pore characteristics of specimens cured for 7 d. The test specimens included two control groups: one containing a rheology modifier (at a dosage of 0.3%) and one without, as well as three mix proportions with c/t ratios of 1:6, 1:8, and 1:10. A total of six groups of specimens were tested. The NMR testing was conducted using a MesoMR23-060H-I nuclear magnetic resonance imaging analyzer manufactured by Suzhou NuMai Analytical Instrument Co., Ltd. (Suzhou, China), with a resonance frequency of 23 MHz. Prior to testing, the instrument was first calibrated using a standard oil sample to establish a quantitative relationship between porosity and the NMR signal intensity. Cylindrical specimens cured for 7 d were removed from the curing chamber and placed in the sample chamber of the NMR analyzer for testing. During the test, the instrument used the Carr–Purcell–Meiboom–Gill (CPMG) pulse sequence to acquire the sample’s transverse relaxation time (T_2_) signal, and the T_2_ spectral distribution curve was obtained through inversion calculations. The peak position distribution and peak area of the T_2_ spectrum reflect the pore size and the volume fraction of the corresponding pores, respectively.

### 2.5. SEM and EDS Testing

A ZEISS EVO18 (Carl Zeiss AG, Oberkochen, Germany) scanning electron microscope (SEM) coupled with an X-ray energy dispersive spectrometer (EDS) was used to observe the microstructure and perform elemental analysis of the fractured specimens. The specimens for SEM testing were taken from representative fragments obtained after uniaxial compression testing. The selected fragments were dried in a constant-temperature oven at 50 °C for 24 h to thoroughly remove any residual adsorbed water from the specimens. Subsequently, the dried specimens were placed in a vacuum sputtering system and subjected to two rounds of carbon sputtering to enhance their electrical conductivity. The treated specimens were then mounted on the specimen stage for observation. The main technical parameters of the SEM were as follows: acceleration voltage of 10–20 kV, maximum magnification of 5000×, and resolution of up to 3 nm. During SEM observation, the integrated EDS energy-dispersive spectrometer probe was used to perform quantitative elemental analysis on selected microareas. EDS analysis yielded characteristic X-ray spectra for elements such as C, O, Si, Ca, Al, Mg, Fe, K, and S.

### 2.6. CT Scan

To characterize the three-dimensional spatial distribution and evolutionary characteristics of internal cracks in CPB at the microscopic scale, computed tomography (CT) scans were performed on failed specimens following uniaxial compression tests. The CT scans were conducted using an EkoVision FF35 high-precision microfocus X-ray 3D scanning system (YXLON International GmbH, Hamburg, Germany). Equipped with a microfocus X-ray source and a high-resolution flat-panel detector, this system is capable of acquiring continuous cross-sectional images of the internal structure without damaging the specimens. During the scanning process, the fractured specimens were securely mounted on the sample stage. The projection data were acquired over a 360° rotation with 2549 projections. The two-dimensional cross-sectional images were subsequently reconstructed from the projection data, and the number of reconstructed slices was determined at the reconstruction stage. After reconstruction, 1700 consecutive 2D cross-sectional images were generated. Each pixel in the images corresponds to an actual physical dimension of 60 micrometers. The CT scans were performed with an acceleration voltage of 130.0 kV and a tube current of 150.0 μA, corresponding to a source power of 19.5 W. The exposure time was 0.50 s, and a 1.0 mm copper filter was used. No frame averaging was applied.

The acquired two-dimensional cross-sectional images are first imported into image processing software for preprocessing, including operations such as image denoising and contrast enhancement, to improve the distinguishability of cracks from the matrix phase. Subsequently, a grayscale threshold-based segmentation algorithm is used to extract and segment the crack structure, with the crack segmentation threshold determined using a sensitivity curve. The threshold for crack segmentation is 0–4346. Remove the pore structure based on the volume differences, and retain only the main crack with the largest volume within the segmented area. Using 3D reconstruction techniques, a series of 2D segmented images are stacked along the scanning direction and reconstructed as voxels to generate a 3D image of the crack structure. Crack parameters are then quantitatively calculated based on the 3D voxel data. The specific analysis parameters include the degree of damage and the 3D fractal dimension. The degree of damage is characterized by the percentage of crack area in the slice relative to the cross-sectional area of the sample. The 3D fractal dimension is determined using the box-counting method. It should be noted that the pixel size in the reconstructed image is 60 μm. Therefore, in this study, only cracks with a width of 60 μm or more are considered.

## 3. Results and Discussion

### 3.1. Mechanical Characteristics

#### 3.1.1. Uniaxial Compression Mechanical Characteristics

**Figure 5:**
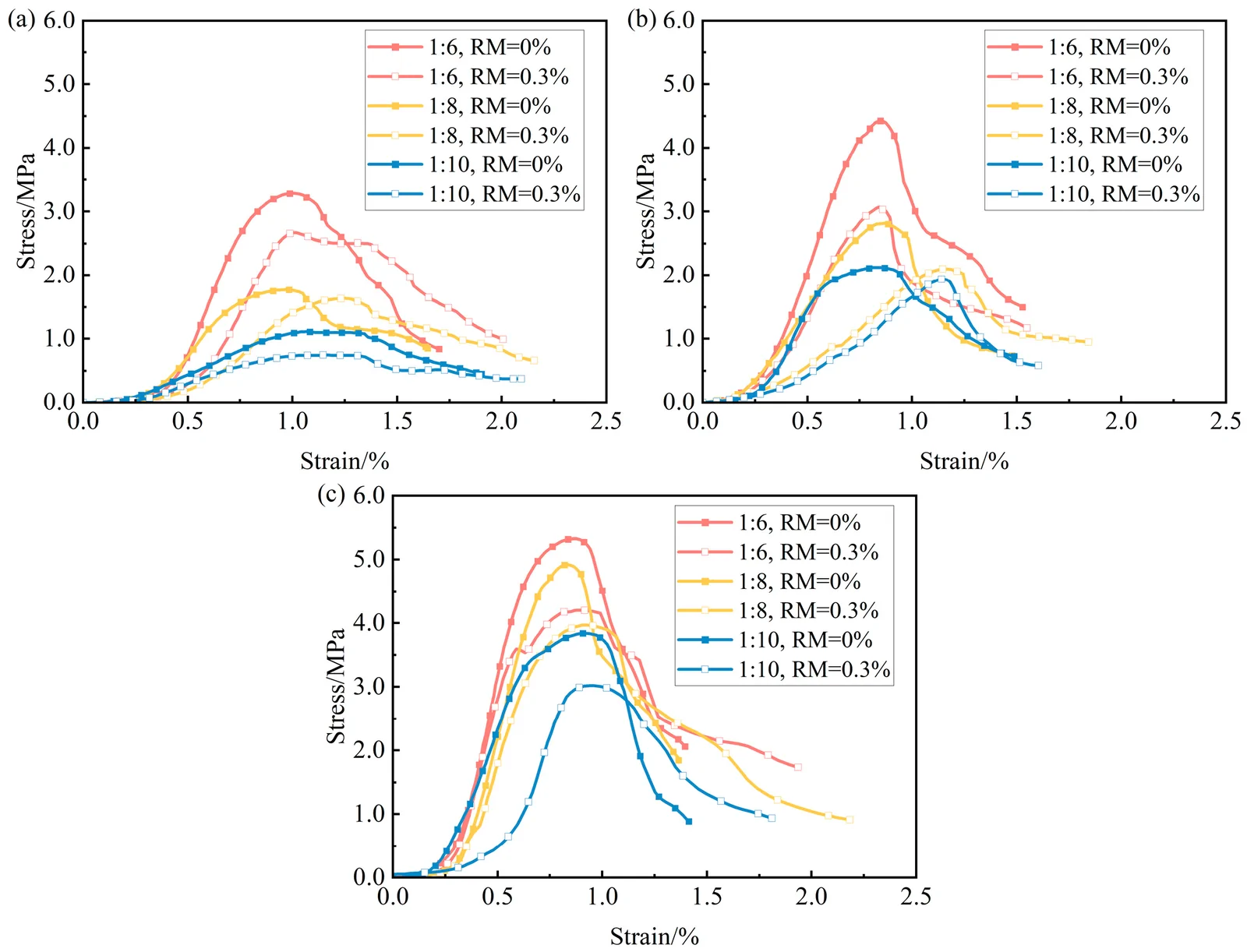
The stress–strain curves of all the CPB samples: (**a**) 7 d; (**b**) 14 d; (**c**) 28 d.

Figure 5 shows the stress–strain curves for all CPB specimens in uniaxial compression tests. In the figure, RM = 0% indicates no rheological modifier, while RM = 0.3% indicates 0.3% rheological modifier. As shown in the figure, the stress–strain responses of all test specimens exhibit typical four-stage characteristics, namely: (i) the pore consolidation stage, (ii) the linearly elastic deformation stage, (iii) the yield stage, and (iv) the post-peak failure stage.

Analysis of the stress–strain curves for different c/t ratios (1:10, 1:8, and 1:6) reveals that as the c/t ratio increases, the slope of the stress–strain curve during the linear elastic stage increases significantly. This indicates that specimens with a higher cementitious material content are better able to resist elastic deformation. Furthermore, specimens with high c/t ratios (especially 1:6) exhibited a steeper stress decay in the post-peak stage. This brittle post-peak behavior indicates a more uniform internal structure and tighter bonding within the specimens. Once the ultimate load is exceeded, cracks propagate rapidly. In contrast, specimens with low c/t ratios (1:8 and 1:10) exhibit a relatively gentle post-peak stress decay, reflecting a more ductile failure mode.

For CPB specimens at different curing ages, the slope of the linear-elastic region of the stress–strain curve gradually increased as the curing time increased from 7 d to 28 d. This indicates that the elastic stiffness increased due to the formation of more hydration products resulting from ongoing hydration reactions. However, the enhanced cementation also led to a more brittle failure response, as evidenced by the faster rate of stress decay in the post-peak phase of the 28 d specimens compared to those at 7 d or 14 d.

Rheology modifier significantly influences the stress–strain behavior of CPB. Compared with samples without rheology modifier, specimens with added rheology modifier exhibited a more pronounced pore consolidation phase during the initial loading stage. This was characterized by greater curvature and a longer strain range in the curve prior to the transition to the linearly elastic region. This suggests that the rheological additive alters the initial particle packing state, potentially forming a slightly looser or more uniformly distributed interparticle pore system that gradually closes during axial loading. The slope of the linearly elastic region decreased after the addition of the rheological additive, and the peak compressive strength showed a significant reduction under all c/t and curing age conditions. This strength reduction can be attributed to the fact that the rheology modifier may have interfered with early hydration kinetics or altered the spatial arrangement of hydration products [33]. Regarding post-peak behavior, specimens with added rheology modifier exhibited a markedly slower stress decay rate after peak stress, indicating enhanced post-peak ductility and residual strength retention.

In summary, analysis of the stress–strain curves indicates that increasing the c/t ratio and extending the curing period can enhance the elastic modulus and peak strength, but also promote brittle failure. The addition of a rheology modifier, on the other hand, reduces strength to some extent, improves ductility, and results in a more pronounced consolidation phase.

**Figure 6:**
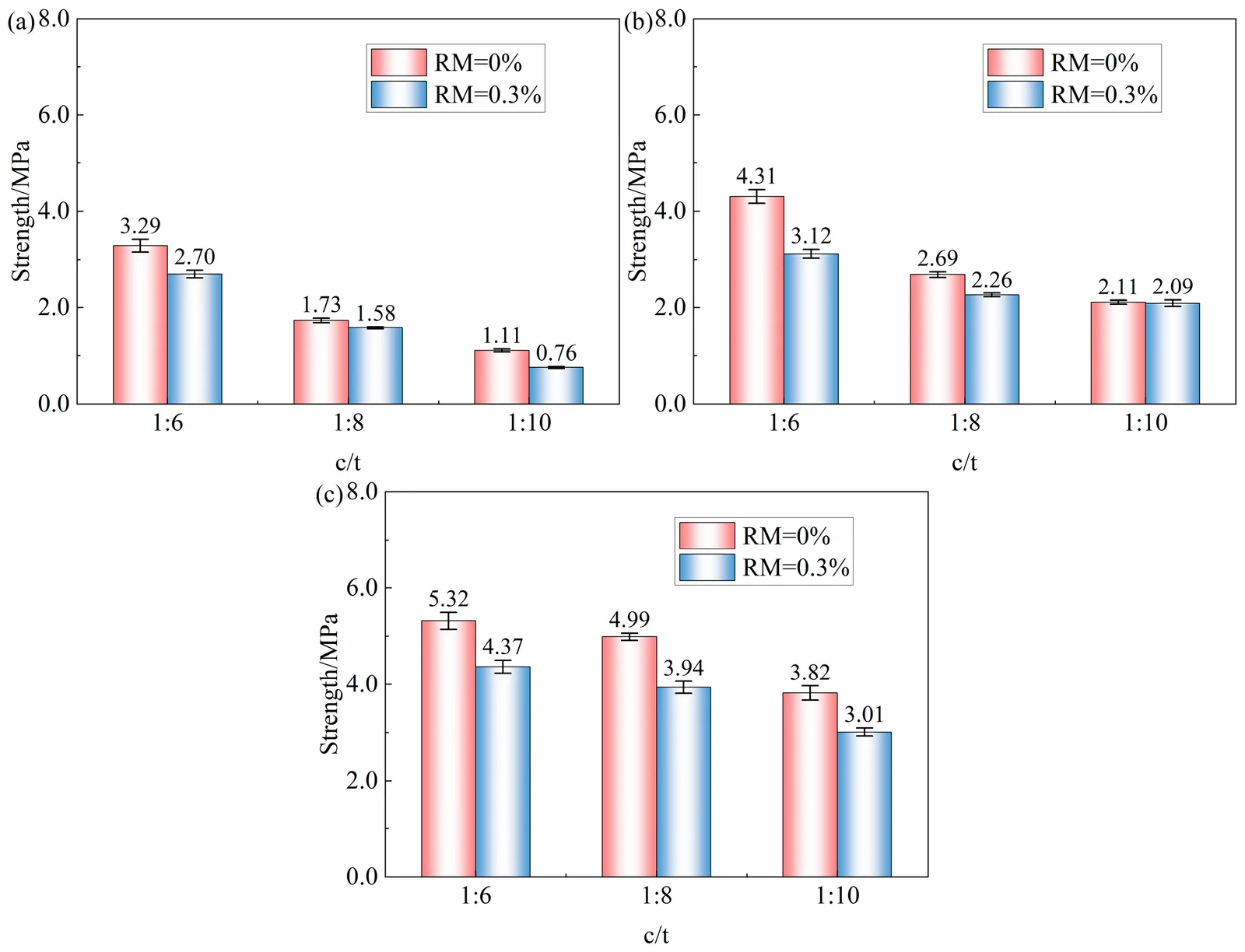
Results for uniaxial compressive strength: (**a**) 7 d; (**b**) 14 d; (**c**) 28 d.

Figure 6 shows the uniaxial compressive strength test results for all test specimens at different curing ages. Figure 6a, Figure 6b and Figure 6c correspond to curing ages of 7 d, 14 d, and 28 d, respectively. Overall, the uniaxial compressive strength of all CPB specimens increased significantly with increasing curing age and c/t ratio. The addition of a rheology modifier resulted in varying degrees of strength reduction for specimens of different mix proportions, but the extent of this reduction varied depending on the c/t ratio and curing age.

Under the same curing age and rheology modifier conditions, the uniaxial compressive strength showed a clear upward trend as the c/t ratio increased. Taking the 28 d specimens without rheology modifier as an example, when the c/t ratio was increased from 1:10 to 1:8, the strength rose from 3.82 MPa to 4.99 MPa, representing an increase of approximately 30.6%; when the c/t ratio was further increased to 1:6, the strength reached 5.32 MPa, representing an increase of approximately 6.6% compared to the 1:8 ratio and approximately 39.3% compared to the 1:10 ratio. A similar increasing trend was observed at 7 d and 14 d curing ages. These results indicate that an increase in the amount of cementitious materials directly provides a greater source of hydration products, thereby promoting their formation. It is worth noting that when the c/t ratio was increased from 1:8 to 1:6, the increase in strength was smaller than the increase observed when the ratio was raised from 1:10 to 1:8, indicating that the rate of strength gain weakens under higher c/t conditions.

Extending the curing period significantly enhances uniaxial compressive strength, and this effect was observed under all mix designs. Taking specimens with a c/t ratio of 1:8 and no rheological additive as an example, the strength at 7 d was 1.73 MPa, rising to 2.69 MPa at 14 d (a 55.5% increase from 7 d), and further increasing to 4.99 MPa at 28 d (an 85.5% increase from 14 d and a 188.4% increase from 7 d). A similar trend of increase was equally evident for c/t ratios of 1:6 and 1:10. It is worth noting that the rate of strength increase from 14 d to 28 d was generally greater than that from 7 d to 14 d, indicating that the hydration of this CPB was not yet complete at the early stage (7 d). The cementitious materials maintained high hydration activity during the period from 14 d to 28 d.

The addition of a rheology modifier resulted in a decrease in uniaxial compressive strength under all mix proportions and curing ages; however, the extent of this decrease varied significantly depending on the c/t ratio and curing age. From the c/t perspective, taking the 28 d curing age as an example, the strength reductions of specimens containing the rheology modifier compared to those without were as follows: at a c/t ratio of 1:6, the strength decreased from 5.32 MPa to 4.37 MPa, a reduction of 17.9%; at 1:8, it decreased from 4.99 MPa to 3.94 MPa, a reduction of 21.0%; at a c/t ratio of 1:10, the strength decreased from 3.82 MPa to 3.01 MPa, a decrease of 21.2%. Overall, the strength-reducing effect of the rheology modifier on specimens with lower c/t ratios (1:8 and 1:10) was slightly greater than that on specimens with higher c/t ratios (1:6), A plausible explanation is that the hydration products in low c/t systems are inherently limited, so that the modifier’s regulation of the floc network structure and liquid-phase viscosity has a relatively greater influence on the continuity of the cementitious network.

The decrease in strength caused by the rheological modifier gradually narrowed as the curing age increased. And this reduction is non-linear. Taking a c/t ratio of 1:6 as an example, the decrease in strength for specimens containing the rheological modifier compared to those without it was 17.9% at 7 d (3.29 MPa → 2.70 MPa), 27.6% at 14 d (4.31 MPa → 3.12 MPa), and at 28 d, it narrowed to 17.9% (5.32 MPa → 4.37 MPa). For a c/t ratio of 1:10, the reduction was 31.5% at 7 d (1.11 MPa → 0.76 MPa) and 21.2% at 28 d (3.82 MPa → 3.01 MPa). At 14 d, the strengths with and without the modifier were 2.09 MPa and 2.11 MPa, respectively, corresponding to a difference of only 0.9%, which is within the range of experimental scatter and is therefore regarded as no significant change rather than a modifier-induced reduction. However, overall, at a longer age (28 d), although the negative effect of the rheology modifier on strength still existed, its impact became more stable compared to the early stages. This phenomenon indicates that as the hydration reaction continues, newly formed hydration products gradually compensate for the structural defects caused by the rheology modifier in the early stages, causing the strength gap between the rheology modifier-containing specimens and the control specimens to stabilize or even narrow slightly.

Based on the above results, the hydration products of cementitious materials are the fundamental source of strength, and their formation is governed by both the c/t ratio and time. Although rheology modifiers improve the rheological properties of fresh slurry, their regulatory effects on the floc network structure and liquid-phase viscosity of the slurry interfere to some extent with the early nucleation and growth environment of hydration products, leading to a reduction in strength. However, given the engineering value of rheology modifiers in improving the pipeline transportability of high-concentration slurries, the resulting strength loss can be compensated for by appropriately increasing the c/t ratio or extending the curing period. In the actual design of CPB systems for mining applications, a comprehensive balance must be struck between transport performance and mechanical properties, and the dosage of rheology modifiers and mix design parameters should be reasonably determined while ensuring the long-term stability of the CPB system.

**Figure 7:**
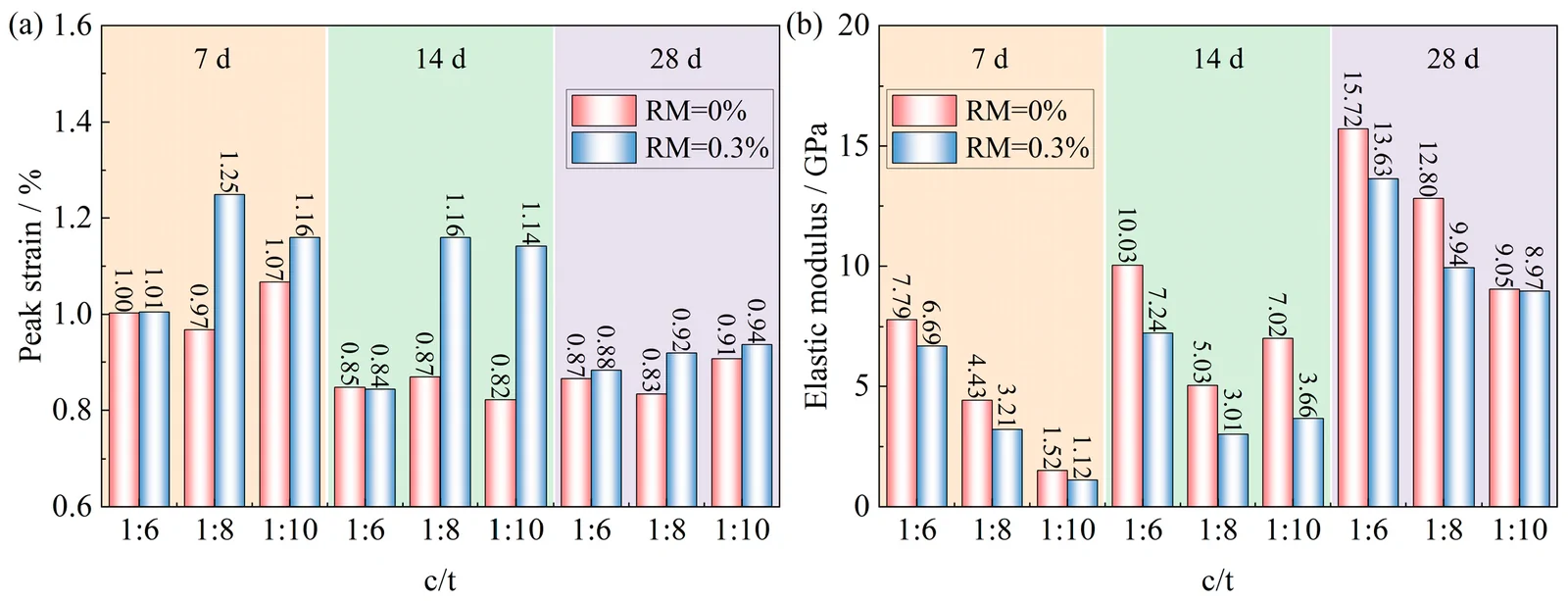
The results of peak strain (**a**) and elastic modulus (**b**) of the sample.

Figure 7a shows the peak strain of CPB specimens under different concentrations of rheological modifiers. From the figure, it can be seen that the peak strain of specimens containing rheological agents is generally greater than that of specimens without rheological agents. This trend is particularly obvious in the 1:6 and 1:10 samples at 7 d and 14 d ages. Taking 7 d, c/t = 1:8 as an example, the peak strain without rheological agent is 0.97%, while with rheological agent it increases to 1.25%, an increase of approximately 28.87%. At c/t = 1:10, the peak strain increases from 1.07% to 1.16%, an increase of approximately 8.41%. However, at the 28 d age, the difference in peak strain between specimens with and without rheological agents significantly narrows. After adding rheological modifiers to the samples, the peak strain increased by 1.9–10.1%. This indicates that as the hydration reaction continues, the hydration products gradually fill the pores and enhance the stiffness of the matrix, and the influence of the rheological agents on the deformation characteristics is partially weakened.

Figure 7b shows the elastic modulus of CPB specimens under different conditions. In terms of the effect of the c/t ratio, the elastic modulus increases significantly as the c/t ratio rises. This trend is consistent with the stiffening effect of hydration products on the matrix. The greater the amount of cementitious material used, the more hydration products are formed. Consequently, the matrix exhibits a stronger resistance to elastic deformation. It should be noted that at 14 days, the 1:10 sample exhibited an unusually high elastic modulus compared to the 1:8 sample, which may be related to variability. Furthermore, the elastic modulus increases with prolonged curing age, reflecting the significant enhancement in matrix stiffness resulting from ongoing hydration. In terms of the effect of rheological modifiers, the elastic modulus of specimens containing rheological modifiers was lower than that of specimens without rheological modifiers under all c/t ratios and curing ages.

#### 3.1.2. Shear Strength

**Figure 8:**
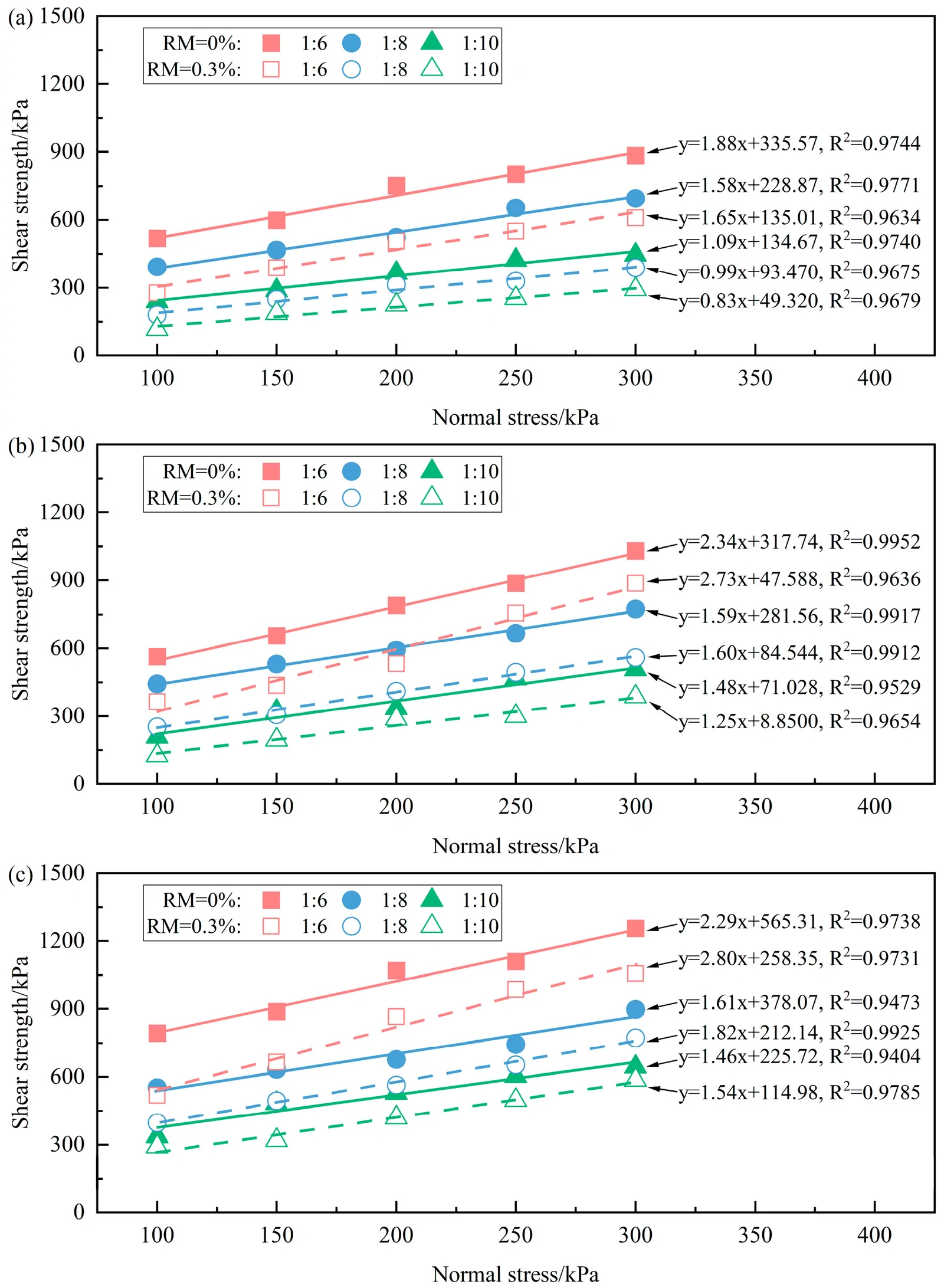
Results for shear strength: (**a**) 7 d; (**b**) 14 d; (**c**) 28 d.

Figure 8 shows the fitted curve of the shear strength of CPB as a function of normal stress. As can be seen from the figure, under all test conditions, the shear strength of CPB exhibits a linear increase with increasing normal stress, and this relationship generally follows the Mohr–Coulomb criterion:

(1) *τ* = *σ*tan*φ* + *c*

Here, *τ* represents the shear strength (kPa), *σ* represents the normal stress (kPa), *φ* represents the angle of internal friction (°), and *c* represents the cohesion (kPa).

The coefficients of determination (*R*^2^) for each curve ranged from 0.9404 to 0.9952. This indicates that the linear fits are highly reliable and that the Mohr–Coulomb criterion is suitable for describing the shear failure behavior of the CPB used in this study. The slope of the fitted straight line represents tanφ, and the intercept represents the cohesion c.

An increase in the c/t ratio significantly enhances shear strength. This effect remains consistent across different curing ages and under different normal stress conditions, and is particularly pronounced under high normal stress conditions. Prolonging the curing age also has a marked enhancing effect on shear strength, although the extent of the increase varies depending on the normal stress conditions. The rate of increase in shear strength with curing age is higher under low normal stress conditions than under high normal stress conditions. This is because, under low normal stress, shear strength primarily depends on the contribution of cohesion, and the enhancing effect of hydration products on cohesion continues to increase with curing age; under high normal stress, frictional resistance accounts for a larger proportion, and its sensitivity to curing age is relatively low.

The addition of a rheology modifier resulted in a decrease in shear strength under all test conditions; however, its effect on the fitting parameters (cohesion and angle of internal friction) followed different patterns, which differed from the negative effects of the rheology modifier on uniaxial compressive strength described earlier. In terms of the fitting parameters, taking 28 d of age and a c/t ratio of 1:6 as an example, the fitting equation for specimens without a rheology modifier is:

(2) *τ* = 2.29*σ* + 565.31, *R*^2^ = 0.9738

(3) *τ* = 2.80*σ* + 258.35, *R*^2^ = 0.9731

The addition of a rheological modifier caused the cohesion to drop significantly from 565.31 kPa to 258.35 kPa, a decrease of 54.3%, while the slope increased from 2.29 to 2.80, corresponding to an increase in the angle of internal friction from approximately 66.4° to approximately 70.4°. Specimens with c/t ratios of 1:8 and 1:10 exhibited similar trends. The above results indicate that the effect of the rheological additive on shear strength is twofold. On the one hand, the rheological additive significantly reduced the cohesion of the backfill, which is consistent with its negative effect on uniaxial compressive strength. On the other hand, the rheological additive did not reduce—and even slightly increased—the angle of internal friction of the CPB, a phenomenon that is difficult to observe in uniaxial compression tests. There may be two reasons for the increase in the angle of internal friction. First, the rheology modifier improves the slurry’s flowability and particle dispersion, allowing tailings particles to rearrange more thoroughly and make closer contact during pouring and curing, thereby forming a particle arrangement more conducive to frictional interlocking. Second, the rheological additive’s regulation of the fine-particle floc network structure reduces the lubricating effect between particles, resulting in more effective interparticle friction and interlocking during shear failure. However, it is worth noting that the increase in the angle of internal friction for specimens containing the rheological additive was relatively limited (approximately 2.6° to 4.0°), which was insufficient to compensate for the strength loss caused by the significant reduction in cohesion. Therefore, shear strength still showed an overall downward trend.

In terms of the decrease in strength under different normal stress conditions (using 28 d and a 1:6 ratio as examples), the decrease for specimens containing a rheological additive was 34.8% at 100 kPa (793.89 → 517.71 kPa), while at 300 kPa, the decrease narrowed to 15.9% (1256 → 1056.18 kPa). The negative effect of the rheological additive is more pronounced under low normal stress conditions, as shear strength at these levels relies primarily on cohesive forces. As normal stress increases, the contribution of frictional resistance grows, while the effect of the rheology modifier on the internal friction angle is minimal or even results in a slight improvement; consequently, the decrease in strength gradually narrows. This finding has significant engineering implications. In high-stress environments such as deep mining, higher normal stress can partially compensate for the loss of cohesion caused by the rheology modifier, bringing the shear resistance of CPB containing the rheology modifier closer to that of the control specimens.

It should be noted that in this study, the normal stress range in the direct shear tests was 100–300 kPa, which is relatively low. Under low normal stress conditions, the specimens have not yet been fully consolidated; the shear swelling effect is significant during the shearing process, and particle rearrangement and interlock are enhanced. This leads to an excessively steep slope of the fitted straight line, resulting in an overestimated apparent angle of internal friction. For some groups, this angle reached 60–70°, which is higher than the 35–50° range commonly reported for CPB materials in the literature [34]. Given the above reasons, the discussion of the friction angle in this paper is based primarily on its relative trends rather than its absolute values.

### 3.2. NMR Test Results

#### 3.2.1. T_2_ Distribution

Convert the T_2_ relaxation time to pore size using the following formula [35]:

(4) $$\frac{1}{T_{2}}=\rho_{2}\left(\frac{S}{V}\right)$$

where *ρ*_2_ is the transverse surface relaxivity and S/V is the surface-to-volume ratio of the pore.

Assuming a cylindrical pore geometry, the pore radius is obtained as [36]:

(5) *r* = 2*ρ*_2_*T*_2_

A surface relaxivity of *ρ*_2_ = 12 μm/s was used for the conversion. By utilizing the correlation between the T2 relaxation time and pore size, the internal pores of the CPB are classified into three categories: micropores (<20 nm), mesopores (20–200 nm), and macropores (>200 nm) [37].

**Figure 9:**
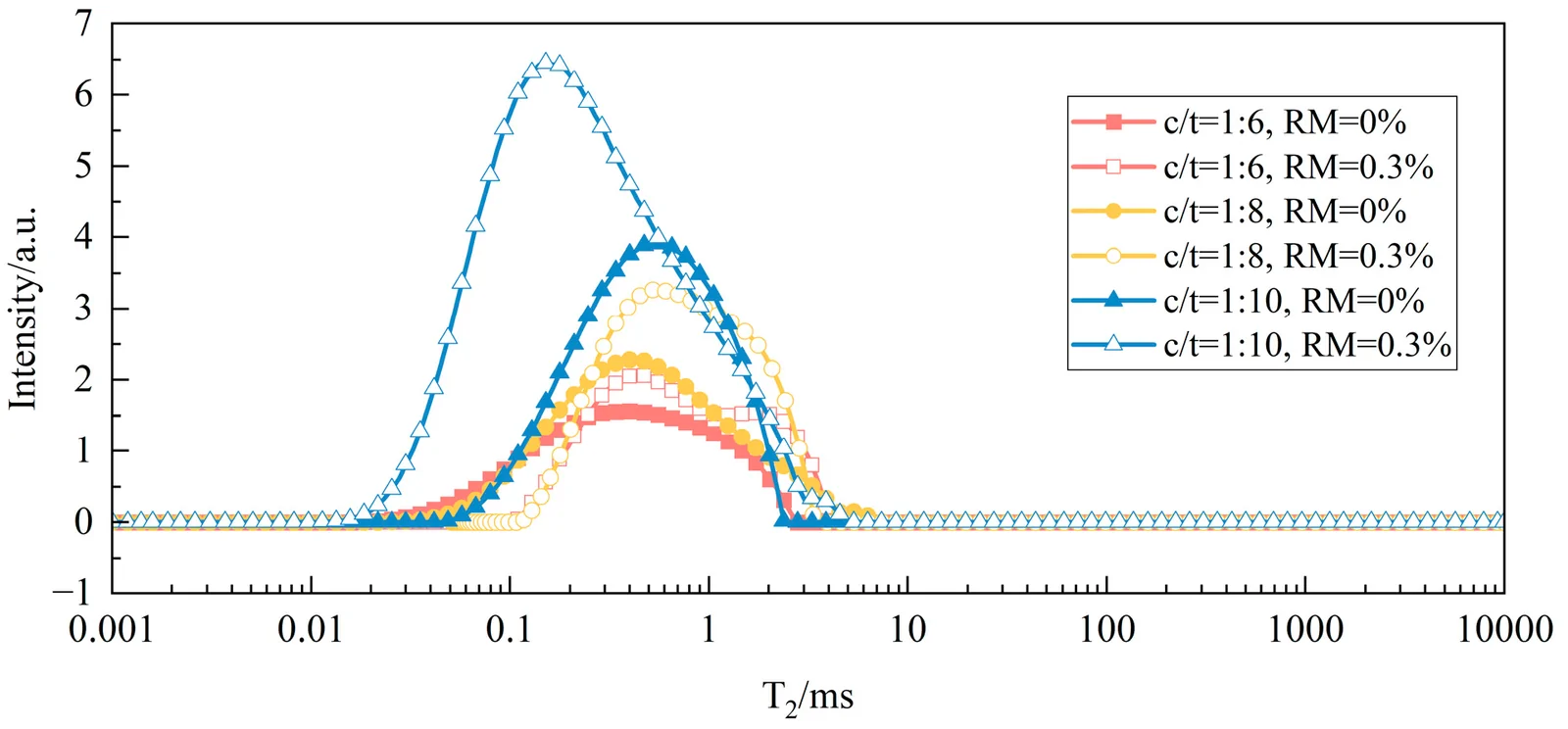
T_2_ curves for different c/t ratios and rheology modifier contents.

Figure 9 shows the distribution curves of the NMR T_2_ spectra of CPB at 7 d of curing under different c/t ratios and different rheology modifier dosages. As shown in the figure, the T_2_ spectra of all samples exhibit a single-peak distribution, with the peak located in the T_2_ = 0.1–1 ms range. This primarily corresponds to gel pores (pore size < 0.01 μm). This indicates that the pore structure of CPB in this study, after 7 d of curing, consists primarily of gel pores. This reflects the microscopic evolutionary characteristics of the early curing stage, during which hydration products are continuously formed and gradually fill the large interparticle pores. No distinct signal peaks corresponding to capillaries or macropores were observed in the T_2_ spectrum.

Regarding the effect of c/t, the peak value of the T_2_ spectrum curve increased significantly as c/t decreased. At c/t = 1:6, the peak value of the main peak in the sample without a rheological additive was approximately 1.56. When c/t was reduced to 1:8, the peak value of the main peak increased to 2.27. When c/t was further reduced to 1:10, the peak value of the main peak reached 3.91. The increase in the peak value indicates an increase in the volume fraction of gel pores within this size range. This is consistent with the microscopic mechanism whereby, under low c/t conditions, the amount of gelling material is reduced, the production of hydration products is insufficient, and the micropores between particles are not fully filled. In terms of the effect of the rheology modifier, under the same c/t ratio, the T_2_ spectral peaks of samples containing the rheology modifier were all higher than those of samples without it. Taking c/t = 1:6 as an example, the peak of the sample containing the rheology modifier was 32.05% higher than that of the sample without it. The same trend was observed for c/t ratios of 1:8 and 1:10. The increase in peak values indicates that the addition of the rheology modifier leads to an increase in the number of micro-pores. This phenomenon may be related to the effect of the rheological modifier on the fresh slurry. By altering the floc network structure and liquid-phase viscosity, the modifier improves flowability; at the same time, it may affect the nucleation and growth environment of the hydration products, which could in turn reduce the filling efficiency and leave more micropores in the hardened matrix [38]. It is worth noting that the negative impact of rheological modifier on the pore structure is more pronounced under low c/t conditions. Comparing the peak differences between samples with and without rheological agents under c/t ratios of 1:6 and 1:10, the increase in peak value is greater for the latter than for the former. This may be because the amount of hydration products is limited in low c/t systems. The pore structure is more sensitive to any disruptive factors. Consequently, the effect of the rheological agent in increasing porosity is further amplified.

#### 3.2.2. Pore Size Distribution

**Figure 10:**
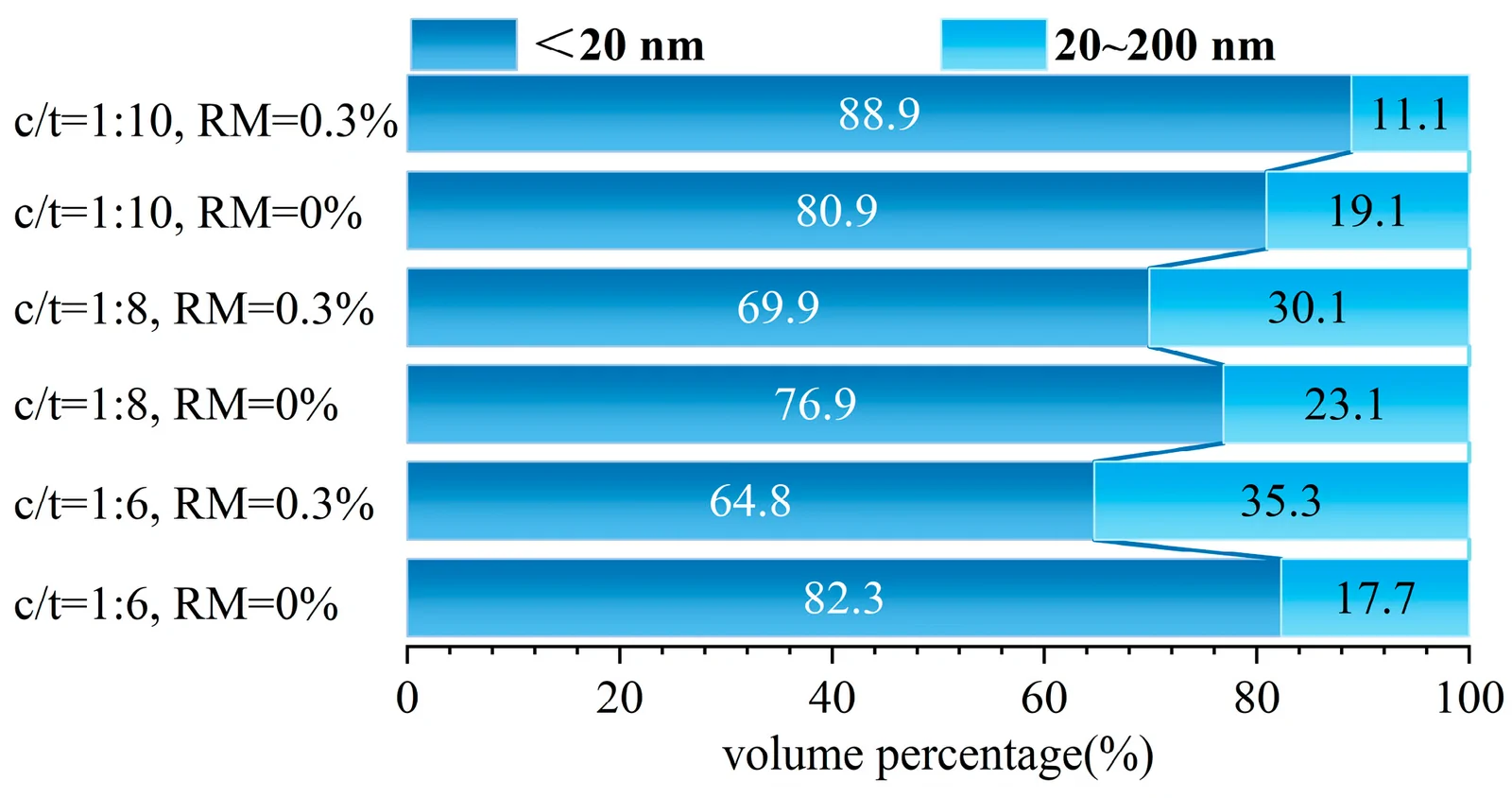
Pore size distribution as a function of different c/t ratios and rheology modifier contents.

Figure 10 shows the pore size distribution results for CPB after 7 d of curing under different c/t ratios and with varying levels of rheological modifier addition. The pores were classified into two size ranges based on pore diameter: less than 20 nm (gel pores) and 20–200 nm (transitional pores/capillary pores), to quantitatively characterize the volume fractions of pores of different sizes.

In terms of the effect of the c/t ratio, in the control group without rheological modifier, as the c/t ratio decreased from 1:6 to 1:10, the proportions of gel pores smaller than 20 nm were 82.3%, 76.9%, and 80.9%, respectively. The proportions of pores in the 20–200 nm range were 17.7%, 23.1%, and 19.1%, respectively. Overall, the effect of c/t variations on the pore size distribution did not follow a monotonic pattern. Pores in this size range were dominant (>75%) under all three c/t ratios. This indicates that, at this curing age, hydration products had effectively filled the large voids between the tailings particles.

In terms of the effect of rheological modifier, under the same c/t conditions, the impact of rheological modifier incorporation on pore size distribution varies depending on the c/t ratio. For c/t = 1:6, in samples containing rheological modifier, the proportion of pores smaller than 20 nm decreased from 82.3% to 64.8% (a 17.5% decrease) compared to samples without rheological modifier, while the proportion of pores between 20 and 200 nm increased from 17.7% to 35.3% (an increase of 17.5%), indicating that the addition of the rheological modifier led to a significant reduction in the proportion of gel pores. For c/t = 1:8, in samples containing the rheological modifier compared to those without it, the proportion of pores smaller than 20 nm decreased from 76.9% to 69.9% (a decrease of 7.0%). The proportion of pores in the 20–200 nm range increased from 23.1% to 30.1% (an increase of 7.0%). These results indicate that the incorporation of the rheological modifier led to a coarsening of the pore structure, with a decrease in the proportion of gel pores and an increase in the proportion of transition pores.

It is worth noting that for a c/t ratio of 1:10, the pore size distribution exhibits an opposite trend. This anomaly may be related to the scarcity of hydration products from the cementitious material under low c/t conditions. Since the total amount of hydration products is limited, the rheological modifier’s improvement in particle dispersion actually causes the fine particles in the tailings to fill the large pores more uniformly, partially offsetting its negative impact on the hydration environment and thus resulting in a relative increase in the proportion of gel pores.

### 3.3. Failure Mode Analysis

#### 3.3.1. Shear Failure Modes

**Figure 11:**
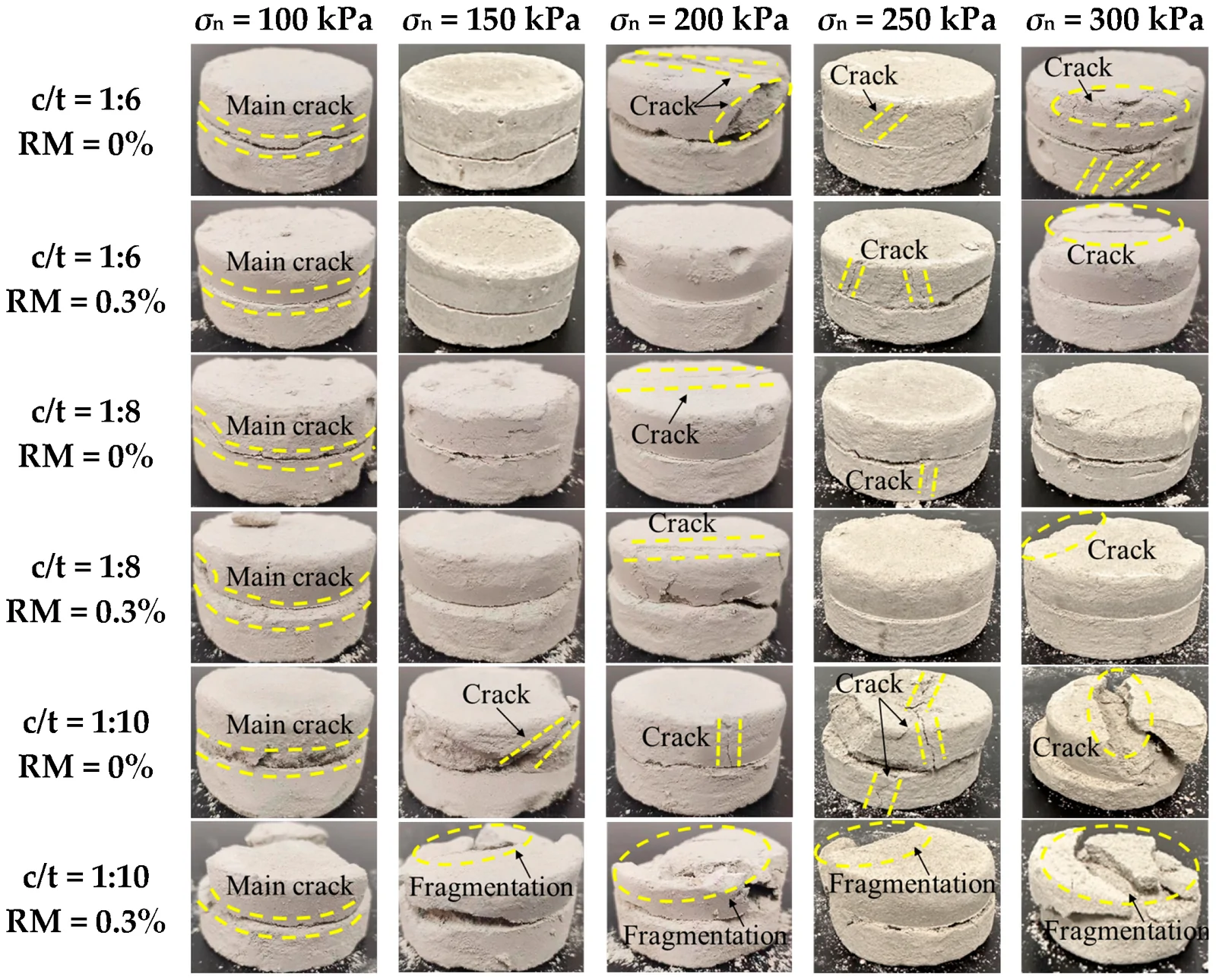
Fracture pattern of a shear test specimen.

Figure 11 shows the shear failure mode of the CPB specimen. After shear failure, all specimens exhibited a primary shear crack in the central region that was approximately parallel to the shear direction; this crack extended horizontally across the entire cross-section of the specimen, exhibiting typical characteristics of shear-slip failure. It is worth noting that, in addition to the primary shear crack, most specimens also developed normal cracks of varying severity on their surfaces, the extent of which was closely related to the test conditions.

The magnitude of the normal stress has a significant effect on the failure mode of the specimens. Under conditions of lower normal stress (100 kPa and 150 kPa), specimen failure primarily manifested as a single through-shear crack. The crack surface was relatively smooth, and the overall integrity of the specimens was good. Relative slip occurs only on both sides of the principal shear plane, and normal tensile cracks rarely appear on the surface. As the normal stress increases to 200 kPa or higher, normal cracks begin to appear on the specimen surface, resulting in a combined shear-tensile failure mode. The formation of normal cracks can be attributed to the significant transverse compressive deformation generated by the relative slippage of particles on both sides of the shear plane under high normal stress. This induces localized tensile stress perpendicular to the shear plane. When the tensile stress exceeds the tensile strength of the aggregate, normal tensile cracks form. From a mechanical perspective, under low normal stress, shear failure primarily overcomes the cohesive forces between particles, resulting in a relatively simple failure surface. Under high normal stress, however, frictional resistance becomes the dominant factor. The shear failure process is accompanied by more intense particle rearrangement and disengagement. The resulting transverse stress components promote the initiation and propagation of normal cracks.

The value of c/t significantly alters the critical normal stress level at which normal cracks appear. For specimens with a c/t ratio of 1:6, normal cracks only appeared clearly under higher normal stress conditions (200 kPa and 250 kPa); In contrast, for specimens with a c/t ratio of 1:10, normal cracks appeared even under lower normal stress conditions (100 kPa and 150 kPa). The underlying mechanism for this difference is that specimens with a high c/t ratio contain a higher proportion of hydration products, resulting in greater interparticle bonding strength. CPB exhibits greater integrity and tensile strength. Consequently, higher normal stresses are required to generate sufficient transverse tensile deformation during shear slippage to induce normal cracks.

Under the same c/t ratio and normal stress conditions, the degree of fragmentation of specimens containing a rheological additive significantly increased after shear failure. Specifically, this manifested as varying degrees of spalling and fracturing at the edges and corners of the specimens; some specimens even split completely along normal cracks. This increased degree of fragmentation can be attributed to the rheological additive causing a reduction in cohesion, which in turn decreases the bonding strength between particles. During the shearing process, cracks are more likely to initiate and propagate at weak bonding interfaces. Ultimately, this results in an increased number of fragments and a higher degree of fragmentation after failure.

#### 3.3.2. Uniaxial Compression Failure Modes Based on CT Scans

**Figure 12:**
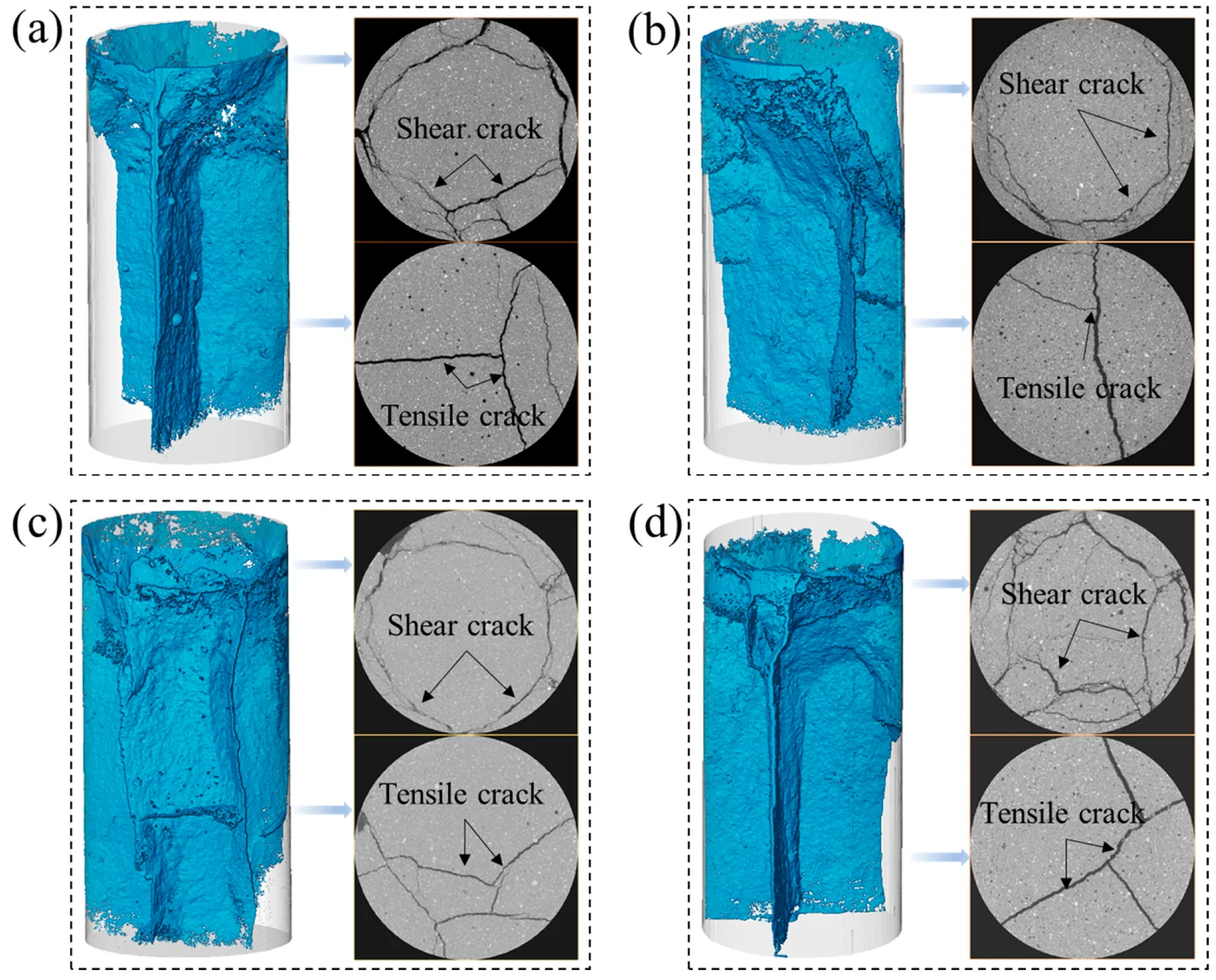
Three-dimensional reconstructed images and 2D slice images of cracks in CPB samples: (**a**) c/t = 1:6, RM = 0%; (**b**) c/t = 1:6, RM = 0.3%; (**c**) c/t = 1:8, RM = 0.3%; (**d**) c/t = 1:10, RM = 0.3%.

Figure 12 shows 3D reconstructed images of the internal crack structure of the CPB specimens, as well as 2D slice images taken at different heights along the specimens. The comparison involves specimens containing a rheology modifier and control specimens without a rheology modifier, both prepared under conditions of a c/t ratio of 1:6 and a curing age of 28 d. It also includes specimens containing a rheology modifier prepared under conditions of c/t ratios of 1:8 and 1:10, with a curing age of 28 d. The 3D reconstructed images reveal that the addition of a rheology modifier did not alter the overall failure mode of the CPB specimens; the spatial distribution characteristics of cracks in both types of specimens were essentially consistent, both exhibiting combined tensile-shear failure. Further examination of the 2D slice images at different heights shows that the top region of the specimen is dominated by annular shear cracks, which develop in a circumferential direction. This indicates that shear stress dominates in this region. In contrast, tensile cracks predominate in the middle and lower regions of the specimen, propagating radially or longitudinally. This reflects the controlling role of tensile stress in these sections. The above results indicate that the rheological additive has a limited effect on the macroscopic failure mode of CPB, and that the spatial distribution of cracks is primarily controlled by loading conditions and specimen geometry.

**Figure 13:**
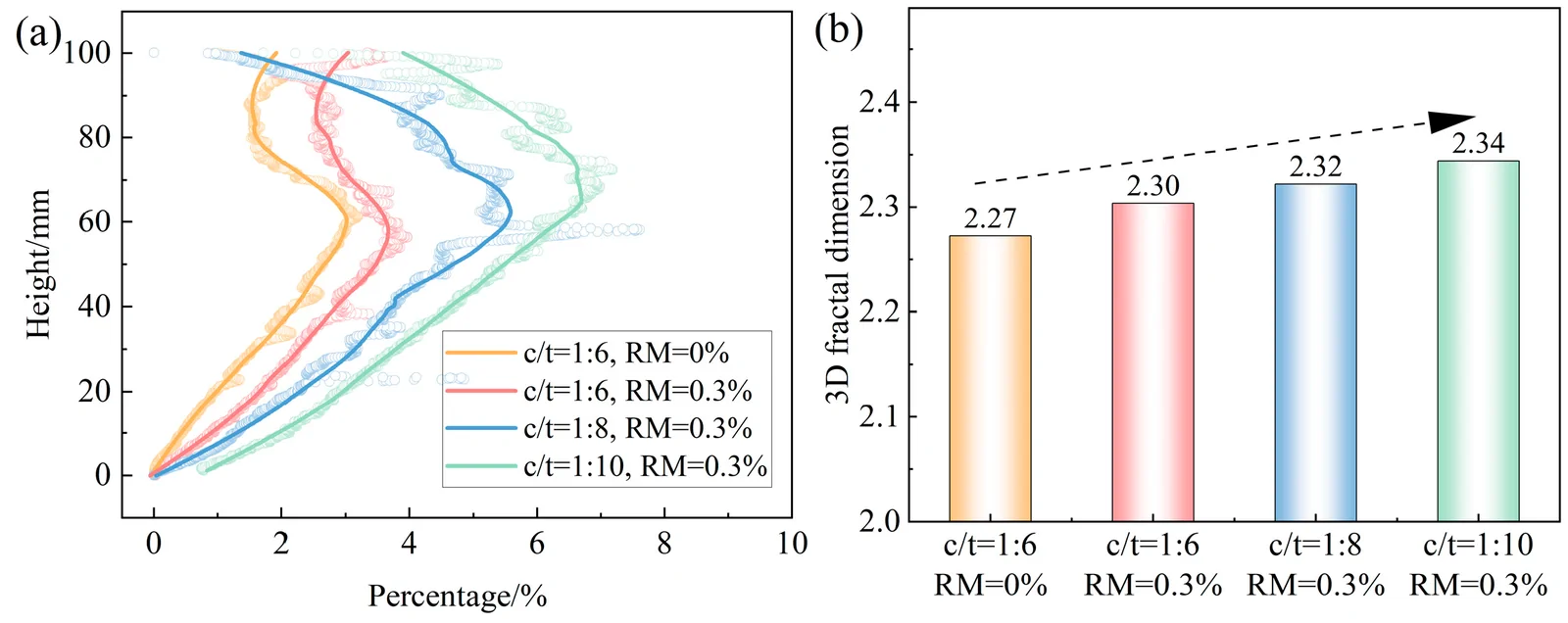
Damage curves (**a**) and 3D fractal dimensions (**b**) of cracks in CPB specimens.

Figure 13 shows the results of a quantitative analysis of the internal crack structure in the CPB specimens. Figure 13a presents the damage curve along the height of the specimen, where the degree of damage is defined as the percentage of the crack area in the 2D slice image at each height relative to the specimen’s slice area. As shown in the figure, the damage curves for all specimens exhibit a trend of first increasing and then decreasing from the bottom to the top. This indicates that cracks primarily develop in the central region of the specimen, with relatively minor damage observed at the top and bottom. A comparison between specimens with and without a rheological additive under c/t = 1:6 conditions reveals that the addition of the rheological additive increases the degree of damage. The peak damage for specimens without a rheological additive was 3.01%, whereas for those with a rheological additive, it was 3.66%, representing an increase of approximately 21.6%. For specimens containing a rheological additive, the damage level increased significantly as the c/t ratio decreased: the peak damage was 5.60% at c/t = 1:8 and further increased to 6.73% at c/t = 1:10, representing increases of 53.0% and 83.9%, respectively, compared to c/t = 1:6. This indicates that a reduction in the amount of cementitious material weakens the bond strength between particles, making CPB more prone to crack initiation and propagation under the same load conditions.

Figure 13b shows the results of the 3D fractal dimension calculations for the crack structure. The fractal dimension reflects the spatial complexity and self-similarity of the crack network. A higher value indicates a more irregular crack morphology and a more complex spatial distribution. As shown in the figure, under a c/t ratio of 1:6, the addition of a rheology modifier increased the fractal dimension of the cracks from 2.27 to 2.30. When the c/t ratio was reduced from 1:6 to 1:10, the fractal dimension of the rheology modifier-containing specimens further increased from 2.30 to 2.34. The trend of increasing fractal dimension is consistent with the pattern of change in peak damage. This indicates that the addition of a rheology modifier and the reduction in the mortar-to-aggregate ratio not only increase the number of cracks but also make the geometric morphology of the crack network more complex and irregular, reflecting an increase in the severity of damage within the CPB.

It should be noted that the CT comparison between specimens with and without the rheological modifier was performed only at a c/t ratio of 1:6. Therefore, the effect of the modifier on crack evolution at c/t ratios of 1:8 and 1:10 cannot be isolated from the present CT data.

### 3.4. Microstructure and Elemental Distribution

**Figure 14:**
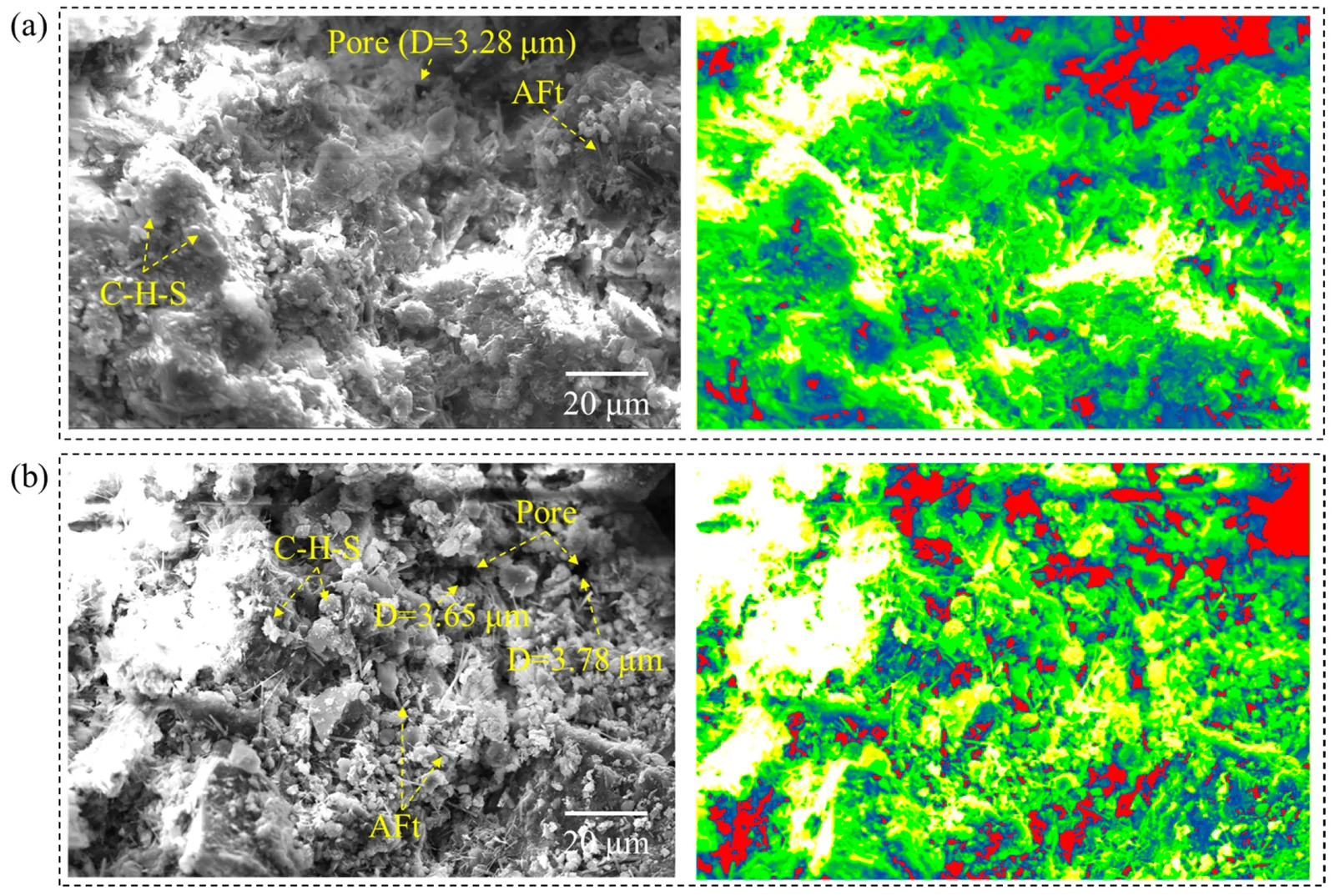
Comparison of SEM images and false-colour processing of CPB: (**a**) RM = 0%; (**b**) RM = 0.3%.

In this study, SEM/EDS analysis was conducted to make a targeted comparison of c and t in order to isolate the effects of the rheological modifier. Figure 14 shows SEM images of samples containing a rheological additive and the control group at a c/t ratio of 1:6, taken at the same magnification. The images presented in this study represent typical microstructures observed in the tested fragments, rather than images of randomly selected areas or specific damage locations. To facilitate a visual comparison and quantitative analysis of the distribution of hydration products and pore development characteristics, the original SEM images were processed using Fiji (ImageJ 2.16.0/1.54p) image processing software to enhance them with false-color rendering, with different colors used to distinguish the matrix from the pores. These colors represent pores and matrix, rather than specific minerals or elements, and the color assignments are arbitrary.

As can be observed in the figure, the types of hydration products within all CPB specimens are essentially the same, with two main categories of typical hydration products present. The first category consists of C-S-H gel distributed in flocculent clusters. These flocculent gels are cross-linked and interlocked, forming a three-dimensional network structure that encapsulates and binds adjacent tailings particles, serving as the primary source of the filler’s strength. The second type consists of ettringite (AFt) in needle-like or rod-like forms. These needle-like or rod-like crystals develop on the inner walls of the voids or interweave within the C-S-H gel network, thereby enhancing the mechanical interlocking effect between particles to a certain extent.

At the same time, the development of micropores was observed in the microstructure of all specimens; these micropores were mostly irregular in shape and were distributed in the interface transition zone between the matrix phase and the hydration product phase, or in the interlocking voids of flocculent gels. The equivalent diameters of the micropores were primarily concentrated in the range of 3–4 μm. As shown in Figure 14a,b, the main difference in microstructure between the two samples lies in the number of micropores. After adding a rheology modifier, the number of microporosity within the CPB increased significantly. This phenomenon may be related to the effect of the rheological modifier on the slurry. The modifier alters the floc network structure and liquid-phase viscosity of the fresh slurry, which may in turn reduce the spatial filling efficiency of the hydration products or change the water migration pathways during curing, leaving more micropores in the hardened matrix [39].

**Figure 15:**
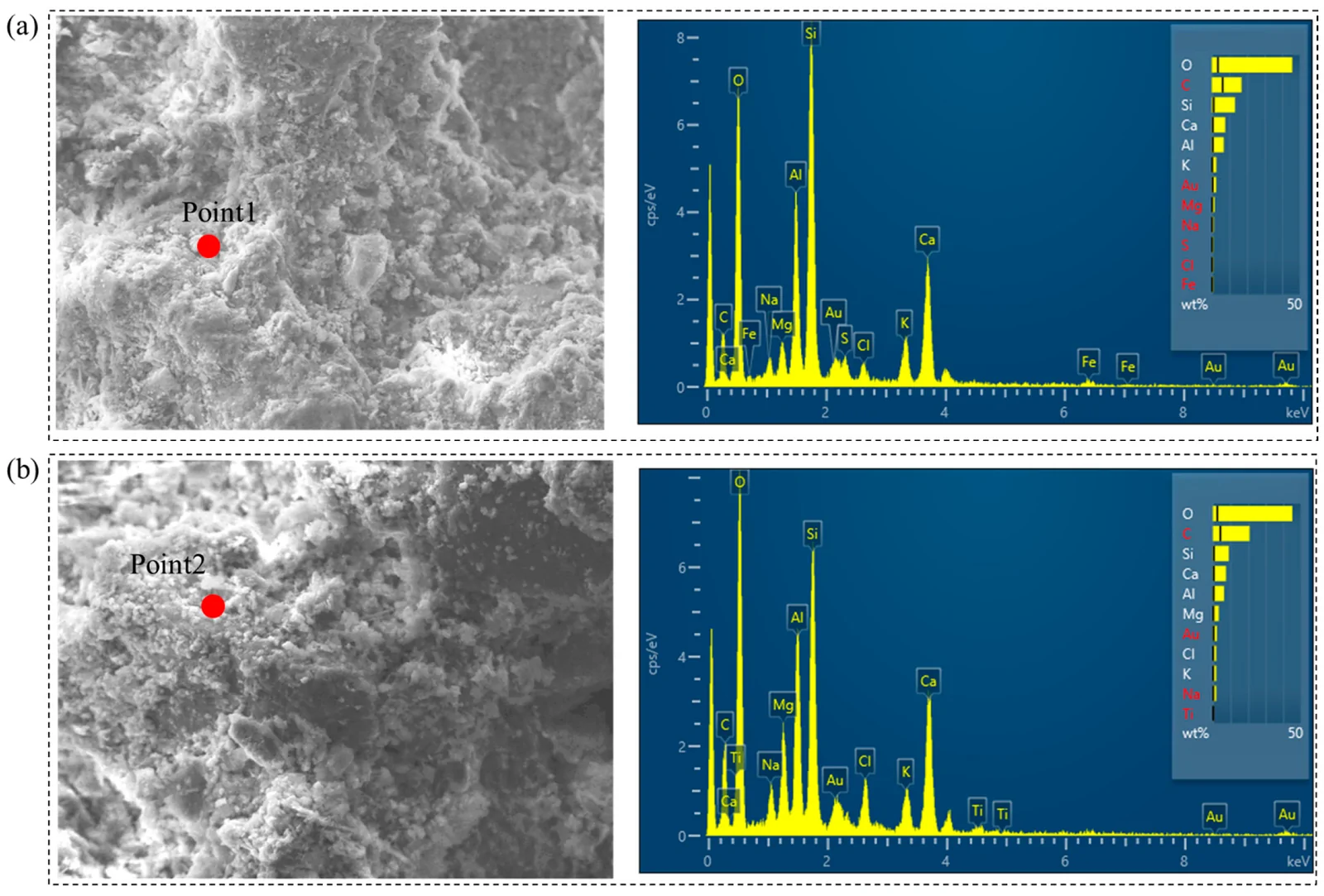
SEM images and EDS point-scan elemental distributions of representative areas: (**a**) RM = 0%; (**b**) RM = 0.3%.

To further determine the chemical composition of the aforementioned hydration products, typical flocculent C-S-H gel regions and acicular and rod-shaped calcium aluminate regions were selected from the SEM images for EDS point-scan analysis. Figure 15 shows the EDS spectra and quantitative elemental analysis results for the corresponding characteristic regions. EDS spectral analysis indicates that the main elements detected in the CPB hydration products are Si, O, Ca, Mg, and Al, accompanied by small amounts of S, K, and Fe. Among these, oxygen (O) accounts for a relatively high mass fraction in all hydration product regions, which is consistent with the characteristic that the hydration products consist primarily of oxides and hydroxides. The high concentrations of Ca and Si reflect the compositional characteristics of the C-S-H gel as the primary hydration product. The EDS analysis is a targeted, semi-quantitative analysis of the two dominant hydration products rather than a statistical characterization of elemental abundance.

It should be noted that EDS provides information on elemental composition rather than on crystal structure or mineral phase. The identification of C-S-H gel and ettringite in this study is therefore based on the combination of morphology observed in the SEM images and the corresponding elemental composition, and does not constitute a quantitative mineralogical determination. The elemental composition of the hydration products is similar in specimens with and without the rheological modifier, which indicates that the modifier does not substantially alter the elemental constituents of the hydration products. However, similar elemental composition does not by itself demonstrate that the hydration kinetics or the crystal development process are identical; the morphology and spatial distribution of the hydration products may still differ. It should be noted that the attribution of the increased micropores to water retention caused by the rheological modifier is a plausible interpretation rather than a directly verified mechanism.

A definitive assessment of phase combinations, hydration kinetics, pore connectivity measurements, and crystal growth requires additional research methods. These were not conducted in this study, and future work will include relevant analyses.

## 4. Conclusions

This study systematically investigated the effects of rheological modifier on the mechanical properties and microstructural evolution of CPB with different c/t ratios (1:6, 1:8, 1:10) at various curing ages (7 d, 14 d, 28 d). Based on uniaxial compression tests, direct shear tests, NMR porosity analysis, 3D CT crack reconstruction, and SEM/EDS microstructural and elemental analysis, the main conclusions are as follows:

(1) Uniaxial compressive strength increases with higher c/t ratios and longer curing periods. The addition of rheological modifier resulted in an 8.7% to 31.5% reduction in uniaxial compressive strength, with this negative effect being more pronounced under a c/t ratio of 1:10 and during the early curing stage (7 d). Analysis of the stress–strain curves indicated that rheological modifier increased the deformation during the pore consolidation stage, reduced the slope of the linearly elastic stage, but significantly slowed the post-peak failure rate.
(2) The results of the direct shear test indicate that the shear strength of CPB exhibits a linear relationship with normal stress and obeys the Mohr–Coulomb criterion. The effect of rheological modifier on shear strength is twofold. Cohesion decreased significantly, while the angle of internal friction increased slightly (by approximately 2.6° to 4.0°). Therefore, under high normal stress conditions, the increase in frictional resistance partially compensates for the loss of cohesion, thereby mitigating the strength-reducing effect of the rheological modifier.
(3) Analysis of shear failure modes indicates that an increase in normal stress causes the failure mode to shift from pure shear failure to a combined shear-tensile failure. An increase in the c/t ratio raises the critical stress threshold for normal crack initiation. The addition of a rheological modifier increases the degree of fragmentation following shear failure. CT 3D reconstruction analysis further revealed that the rheological modifier did not alter the overall tensile-shear composite failure mode of CPB under uniaxial compression. However, it increased the extent of crack damage and the fractal dimension of the cracks, with crack development being more pronounced under low c/t conditions.
(4) Analysis of the NMR T_2_ spectrum indicates that the pore structure of CPB cured for 7 d consists primarily of gel pores. As the c/t ratio decreases, the T_2_ spectrum peak significantly increases; the addition of a rheological modifier also leads to an increase in the T_2_ spectrum peak, indicating that the rheological modifier increases the number of micropores. SEM observations confirmed that the interior of CPB primarily consists of two types of hydration products: flocculent C-S-H gel and acicular and prismatic ettringite. The diameters of the micropores were concentrated in the range of 3–4 μm. The number of micropores increased significantly after the addition of the rheological modifier. However, the basic chemical composition of the hydration products remained unchanged. The elemental composition of the hydration products was similar in specimens with and without the rheological modifier; however, this observation does not by itself establish that the hydration kinetics or crystal development are identical, and a definitive assessment would require phase-specific and time-resolved analyses.

It should be noted that this study has several limitations. The normal stress range used in the direct shear tests was relatively low, so the absolute values of the friction angle should be treated with caution. Absolute porosity and cumulative pore volume were not calibrated. The mechanical and microstructural data were obtained from different specimens, and their relationship is therefore qualitative. The mechanism by which the modifier increases microporosity and reduces strength is inferred from multi-scale evidence and requires further verification through hydration kinetics or pore connectivity measurements. These aspects are recommended for future work.

## Figures and Tables

**Table 1:** Mix proportions and curing schemes of CPB specimens.

Group ID	c/t	Rheology Modifier/wt.%	Curing Time/d	Sample Size/mm
1	1:6	0	7/14/28	50*25 mm 50*100 mm
2	1:8	0		
3	1:10	0		
4	1:6	0.3		
5	1:8	0.3		
6	1:10	0.3		

## Data Availability

The original contributions presented in this study are included in the article. Further inquiries can be directed to the corresponding authors.
